# Research progress of mitophagy in chronic cerebral ischemia

**DOI:** 10.3389/fnagi.2023.1224633

**Published:** 2023-08-03

**Authors:** Mayue Yu, Manqing Zhang, Peijie Fu, Moxin Wu, Xiaoping Yin, Zhiying Chen

**Affiliations:** ^1^Department of Neurology, Clinical Medical School of Jiujiang University, Jiujiang, Jiangxi, China; ^2^Jiujiang Clinical Precision Medicine Research Center, Jiujiang, Jiangxi, China; ^3^School of Basic Medicine, Jiujiang University, Jiujiang, Jiangxi, China

**Keywords:** chronic cerebral ischemia, stroke, mitochondrial autophagy, oxidative stress, treatment

## Abstract

Chronic cerebral ischemia (CCI), a condition that can result in headaches, dizziness, cognitive decline, and stroke, is caused by a sustained decrease in cerebral blood flow. Statistics show that 70% of patients with CCI are aged > 80 years and approximately 30% are 45–50 years. The incidence of CCI tends to be lower, and treatment for CCI is urgent. Studies have confirmed that CCI can activate the corresponding mechanisms that lead to mitochondrial dysfunction, which, in turn, can induce mitophagy to maintain mitochondrial homeostasis. Simultaneously, mitochondrial dysfunction can aggravate the insufficient energy supply to cells and various diseases caused by CCI. Regulation of mitophagy has become a promising therapeutic target for the treatment of CCI. This article reviews the latest progress in the important role of mitophagy in CCI and discusses the induction pathways of mitophagy in CCI, including ATP synthesis disorder, oxidative stress injury, induction of reactive oxygen species, and Ca^2+^ homeostasis disorder, as well as the role of drugs in CCI by regulating mitophagy.

## 1. Introduction

Chronic cerebral ischemia (CCI) is considered low-efficiency functional congestion caused by long-term vascular disease or circulatory disorders. It plays a crucial role in cerebrovascular and neurodegenerative diseases and can lead to diseases such as vascular dementia (VD) and Alzheimer’s disease (AD) (Gao, 2018; Ciacciarelli et al., 2020; Li et al., 2022). Studies have shown that symptoms such as headache and dizziness caused by CCI are reversible when cerebral blood supply insufficiency is relieved (Calabrese et al., 2016). Active secondary prevention can reduce ischemic stroke recurrence by approximately 80% (Hankey, 2014). In contrast, the risk of acute stroke, vascular cognitive impairment, and dementia increases if the ongoing decline in cerebral blood flow is not corrected in a timely manner (Liao et al., 2016; Rajeev et al., 2022). According to the Global Burden of Disease (2020) statistical report, the incidence of ischemic stroke worldwide accounted for 64.8%, with a prevalence of 76.5%.

In addition to serving, as a source of bioenergy, mitochondria directly regulate programmed cell death (Kislin et al., 2017). Mitochondrial damage has been reported as a pathological mechanism leading to ischemic neuronal death (Anzell et al., 2018). Autophagy, an intracellular lysosomal degradation pathway, can be classified into canonical and non-canonical pathways. Autophagy processes have been shown to include autophagosome induction and formation and autophagic flux (Xie and Klionsky, 2007). Autophagic flux consists of autophagosome trafficking and fusion with lysosomes to form autophagolysosomes, in which autophagic contents are broken down (Xie and Klionsky, 2007). Mitophagy is the process of targeting damaged or dysfunctional mitochondria and delivering them to lysosomes for degradation, complete self-renewal, and maintaining homeostasis (Pickles et al., 2018). Several CCI-induced neurodegenerative diseases, including VD and AD, are significantly influenced by mitophagy (Arun et al., 2016). Mitochondrial autophagy has a dual function. Its negative effect is the induction of neuronal death (cytodestructive autophagy), while its protective function is to prevent the accumulation of damaged mitochondria (cytoprotective autophagy) (Zhang et al., 2021). If its protective properties can be used effectively, the regulation of mitochondrial autophagy may be a valuable therapeutic target. However, compared to research on the mechanism of mitophagy in acute cerebral ischemia, insufficient research has been conducted on this mechanism in CCI nationally and internationally (Nguyen et al., 2018; Wang et al., 2021; Li et al., 2023). In light of these circumstances, this study aimed to explore the mechanism of mitophagy and its function in CCI and to offer new suggestions for clinical management.

## 2. Induction pathway of mitophagy after chronic cerebral ischemia

**FIGURE 1:**
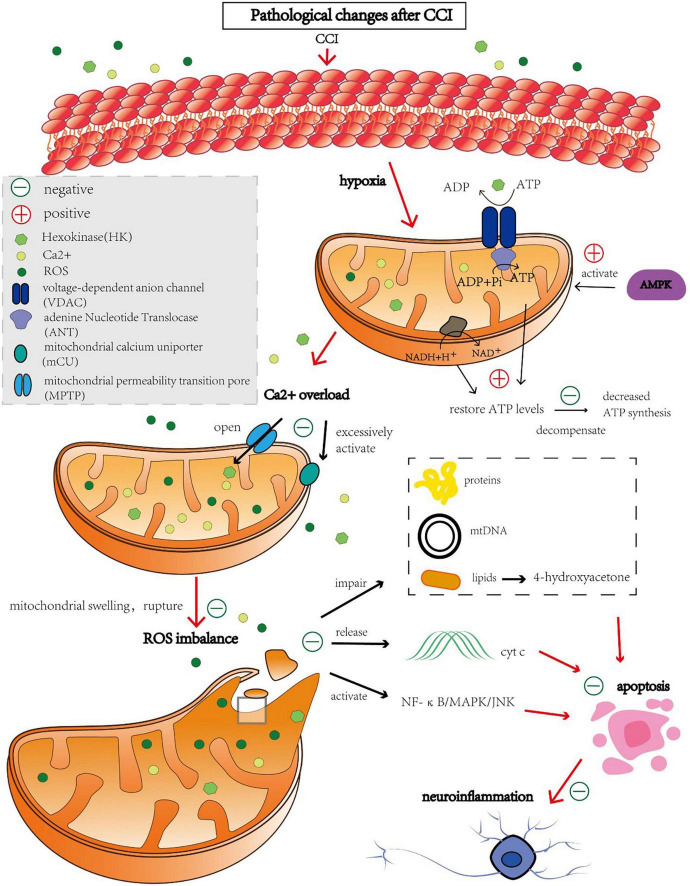
Molecules causing mitochondrial autophagy after CCI and regulatory protective targets.

The brain uses more oxygen than any other organ and is highly metabolically active. Although it makes up only 2% of the human body by weight, brain tissue delivers 25% of the glucose and approximately 20% of the oxygen required by the body (Siwicka-Gieroba et al., 2022). Brief periods of ischemia and hypoxia can seriously harm the brain. Mitochondria play a crucial role in cellular energy stations such as ATP production, reactive oxygen species production, Ca^2+^ homeostasis, and apoptosis (Tang et al., 2016). A detailed diagram of this mechanism is shown in Figure 1. As a result, the normal physiological activities of brain cells are closely related to the normal function of mitochondria.

### 2.1. ATP synthesis disorder

Metabolic disorders are believed to be the first causal factor of CCI. Following cerebral ischemia, partial pressure of oxygen in the brain decreases. Most aerobic oxidation pathways switch to anaerobic glycolysis, and adenosine monophosphate-activated protein kinase (AMPK) is activated. Active AMPK phosphorylates multiple downstream substrate proteins, inhibits the biosynthetic pathway of ATP consumption, and negatively regulates ATP regeneration to restore cellular energy levels as much as possible (Andjelkovic et al., 2019). However, as the duration of ischemia increases, this negative feedback cannot compensate for the loss of mitochondrial energy and downregulation of the expression of proteases involved in oxidative phosphorylation complexes, such as nicotinamide adenine dinucleotide dehydrogenase and cytochrome oxidase, which reduce ATP synthesis (He et al., 2012).

### 2.2. Oxidative stress injury

Mitochondrial permeability transition pore (MPTP) is a non-specific voltage-dependent special protein complex that crosses the mitochondrial outer membrane and controls mitochondrial permeability (Halestrap, 2009). In the physiological state, MPTP is switched off. However, the MPTP is open during ischemia, which is triggered by Ca^2+^ overload and elevated oxidative stress in the mitochondrial matrix (Kushnareva and Sokolove, 2000; Zhao et al., 2019). The opening of the MPTP leads to an increase in mitochondrial permeability, which allows solutes such as water, macromolecules, and ions to freely enter the mitochondrial matrix, resulting in mitochondrial swelling, outer membrane rupture, and the release of large amounts of reactive oxygen species (ROS) (Krasnikov et al., 2005). In addition, increased mitochondrial permeability also leads to the loss of membrane potential, which in turn lowers cellular mitochondrial ATP levels, enhances intracellular Ca^2+^ concentration, and activates the endogenous apoptotic pathway, thereby inducing neuronal damage caused by ischemia and hypoxia (Zorov et al., 2000; Broughton et al., 2009).

Nuclear respiratory factor 2 (Nrf2) is a key transcription factor of antioxidants (Hannan et al., 2020). When cells undergo oxidative stress, Nrf2 is activated, enters the nucleus, binds to promoters of antioxidant response genes and promotes their transcription and expression (Yen et al., 2016). These genes include superoxide dismutase, glutathione peroxidase, and glutathione S-transferase, which scavenge free radicals and other oxidative substances, reducing damage from oxidative stress in cells (Yang et al., 2019). URB597 alleviates ischemic cerebrovascular disease by activating the Nrf2 pathway, reducing mitochondrial oxidative stress and inflammation (Wang et al., 2022).

### 2.3. Induction of ROS

When entering equilibrium with the antioxidant system, ROS cause minimal damage under typical circumstances (Takizawa et al., 1998). However, after CCI, the activity of the respiratory chain enzyme complex is inhibited, mitochondrial respiratory dysfunction occurs, and excessive ROS (Yu et al., 2014). Excessive ROS damage to proteins, mtDNA, and lipids leads to apoptosis, neuroinflammation, and destruction of the blood-brain barrier in the ischemic brain (Shirley et al., 2014). Excess ROS levels induce apoptosis through lipid peroxidation. In rats with cerebral ischemia, 4-hydroxyacetone, a by-product of lipid peroxidation, increases and induces axonal damage and oligodendrocyte apoptosis (Mccracken et al., 2000; Matsuda et al., 2009). ROS can also lead to cell apoptosis by releasing cytochrome c (Cyt c), improving mitochondrial permeability and activating the NF-κB/MAPK/JNK pathway (Kim et al., 2006, 2010). In particular, Cyt c is a soluble protein anchored to the inner mitochondrial membrane, and upon release from mitochondria, it triggers a cascade of apoptotic signaling, which typically peaks after ischemia in Cyt c release (Hüttemann et al., 2011; Tajiri et al., 2016). Mammalian target of rapamycin (mTOR) is an important cell signal transduction pathway, which is involved in the regulation of cell growth, metabolism and autophagy (Saxton and Sabatini, 2017). Activation of mTOR signaling pathway can inhibit ROS production. Ethidium bromide, for example, induces mitochondrial clearance through the autophagy pathway (Luo et al., 2013). However, inhibition of mTOR with rapamycin preserved mitochondrial membrane potential and reduced the production of ROS (Nacarelli et al., 2014). In addition, sertraline is a selective serotonin reuptake inhibitor (SSRI) that regulates AMPK-mTOR signal-mediated autophagy by targeting the mitochondrial voltage-dependent anion channels 1 protein (VDAC1) (Hwang et al., 2021). To sum up, there is a complex interaction between mTOR, ROS and mitophagy. Most importantly, Autophagy is regulated in the nervous system by activating ROS and mediating the Akt-mTOR signaling pathway (Gao et al., 2019; Liu et al., 2019a,b).

### 2.4. Ca^2+^ homeostasis and post-apoptotic induction

After CCI, cells are unable to maintain a negative membrane potential due to the lack of ATP, and neuronal depolarization results in an influx of calcium ions into the cell (Gouriou et al., 2011). An excessive increase in calcium ion concentration activates the mitochondrial calcium uniporter (mCU) in cells, which changes mitochondrial permeability, impairs its ability to generate ATP, and leads to the release of proapoptotic factors (Gouriou et al., 2011). Preclinical research is currently being conducted on medications that block mCU, such as Ru360 (García-Rivas et al., 2006). Even partial inhibition of calcium uptake prevents mitochondrial depolarization, the opening of large mitochondrial channels, and cytochrome c release.

Hexokinase is a six-carbon sugar phosphorylase involved in glycolysis, from which ATP is produced (Tan and Miyamoto, 2015). Furthermore, after CCI, the levels of VDAC, especially VDAC1, have been found to decrease and the interaction between VDAC1 and hexokinase has been reduced. These changes may result in a reduction in ATP/ADP exchange and affect the transport of small-molecule metabolites required for oxidative phosphorylation to mitochondria, thus inhibiting respiration and affecting mitochondrial energy supply and mitochondrial-mediated apoptosis (He et al., 2012).

## 3. The role of mitophagy in CCI

**FIGURE 2:**
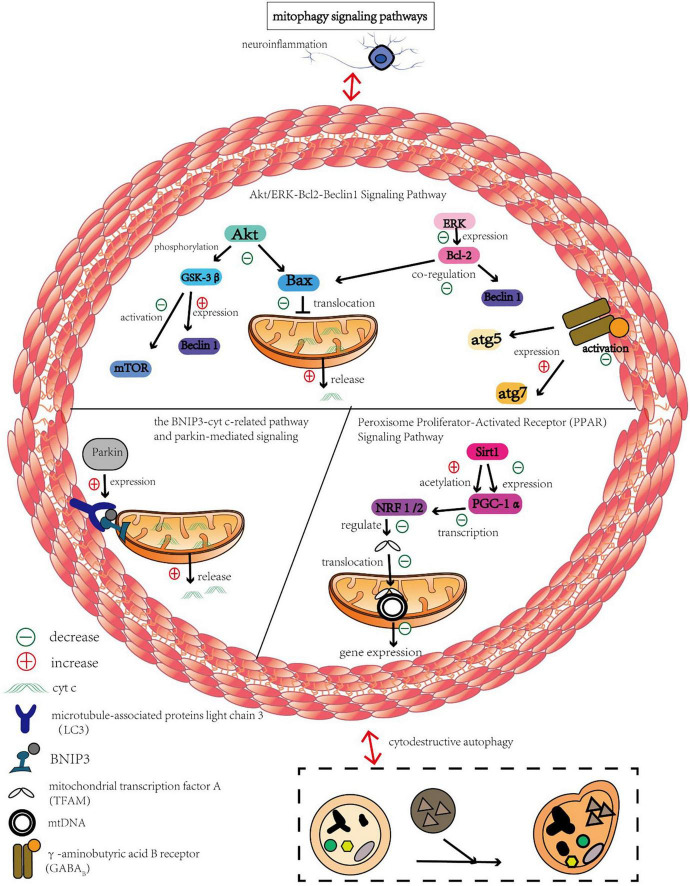
Mitochondrial autophagy pathway in CCI.

The regulation of mitophagy has a wide range of potential applications for the treatment of CCI and the defense of injured brain tissue, as mitophagy mediates a number of signaling pathways that play an important role in the disease. Reviewing prior regulation of mitophagy signaling pathways and regulatory variables has provided information on the study and development of new medications. The mitochondrial autophagy pathway in CCI is shown in Figure 2.

### 3.1. Parkin pathway

Mitophagy is initiated during neuronal apoptosis following CCI through the BNIP3-Cyt c-related pathway and parkin-mediated signaling (Su et al., 2018). After CCI induction, Parkin and BNIP3 expression increased, and Cyt c was released from the mitochondria into the cytoplasm; however, the first two phenomena were significantly attenuated after treatment with the autophagy inhibitor 3-MA. Similarly, URB597 (an orally biocompatible inhibitor of fatty acid amide hydrolase) treatment significantly reversed the increase in Beclin-1, parkin, and BNIP3 protein expression and the decrease in autophagy-related proteins after CCI (Su et al., 2018). Autophagy consists of three major sequential steps: sequestration, transport, and degradation (Mizushima, 2007). During degradation, autophagosomes and their cargo are degraded by lysosomal hydrolases, and lysosomal dysfunction can lead to accumulation of autophagosomes (Mizushima et al., 2008). Some researchers have argued that this accumulation should be treated as an abnormally excessive form of autophagy (Su et al., 2018). Further, the beneficial effects of URB597 on chronic ischemic brain injury occur by inhibiting impaired autophagic degradation and disruption of the Beclin-1/Bcl-2 complex, followed by severing BNIP3-Cyt c and parkin-mediated mitophagy; this ultimately prevents abnormal excessive autophagy and mitophagy (Su et al., 2018).

### 3.2. Peroxisome proliferator-activated receptor (PPAR) signaling pathway

The biological function of PPAR depends on the coactivation of PPAR-γ coactivator 1α (PGC-1α) (Haemmerle et al., 2011). PGC-1α is a master transcription factor in the regulation of antioxidant enzymes, clearance systems, and mitochondrial biogenesis (Kaarniranta et al., 2020). Once activated by phosphorylation or deacetylation, PGC-1α activates the transcription of NRF 1 and 2, which regulates mitochondrial transcription factor A (TFAM) (Li et al., 2017). TFAM then translocates to the mitochondrial matrix and stimulates mtDNA replication and mitochondrial gene expression (Tang, 2016). The upstream transcription factor Sirt1 regulates PGC-1α by increasing its expression and decreasing its acetylation (Iwabu et al., 2010). Nicotinamide adenine dinucleotide (NAD), a substrate of Sirt1 that regulates Sirt1 expression, improves cognitive function and reduces neuroinflammation in *in vivo* and *in vitro* CCI models (Mouchiroud et al., 2013). Furthermore, these therapeutic effects were associated with mitochondrial protection and inhibition of ROS by activating the Sirt1/PGC-1α pathway (Zhao et al., 2021).

### 3.3. Akt/ERK-Bcl2-Beclin-1 signaling pathway

Akt activation enhanced GSK-3β phosphorylation, leading to mTOR activation, and the autophagic protein Beclin-1 expression was significantly downregulated, inhibiting cell cytodestructive autophagy (Wang R. C. et al., 2012; Liu et al., 2015). Akt phosphorylation prevents Bax translocation to mitochondria and inhibits Cyt c release as well as destructive autophagy, attenuating CCI-induced neuronal injury (Sadidi et al., 2009; Castillo et al., 2011). Meanwhile, ERK activation upregulates Bcl-2 expression, which negatively regulates destructive autophagy through a combination of Beclin-1 and Bax (Subramanian and Shaha, 2007). Activation of the γ-aminobutyric acid B receptor (GABA_B_) can attenuate CCI-induced increases in atg5 and atg7 expression and inhibit cytodestructive autophagy and neuronal apoptosis (Lindqvist et al., 2014). Baclofen-induced ERK1/2 phosphorylation can accelerate cytoprotective autophagy by moderately increasing the expression of Beclin-1. Activation of GABA_B_ receptors improves the surface expression of the GABA_A_ receptor α1 subunit, leading to the downregulation of astrocytes and neurons surface and mitochondrial expression, which in turn enhances cytoprotective autophagy (Liu et al., 2015).

### 3.4. Mitochondrial membrane ATP-sensitive potassium channel (mitoKATP)

The opening of mitoK_ATP_ channels is related to potassium uptake from the mitochondrial matrix and maintains the volume of the mitochondrial matrix by reducing the Ca^2+^ load. Reduced mitochondrial Ca^2+^ load can inhibit MPTP opening, prevent ROS production in the mitochondria, and inhibit excitatory oxidative stress and cell death (Fornazari et al., 2008). mitoK_ATP_ consists of two subunits, Kir6.1, 6.2, and SUR1 or SUR2 (Zhou et al., 2010). Chronic intermittent hypobaric hypoxia (CIHH) can upregulate the protein expression of Kir6.2 and SUR1 in the mitochondria of the hippocampal CA1 region induced by ischemia, thus improving learning and memory dysfunction induced by ischemia in the hippocampal CA1 region. Additionally, CIHH alleviates delay neuronal death (DND) by maintaining mitoK_ATP_ activity, thus inhibiting Cyt c-induced apoptosis (Zhang et al., 2016).

## 4. Role of mitophagy regulating drugs in CCI

Bilateral common carotid artery occlusion (2VO) has been used to create a CCI animal model in most trials to investigate the underlying mechanism (Du et al., 2017). The pathogenic role of cerebral hypoperfusion in neurodegenerative diseases can be understood from data collected using a rat 2VO model (Farkas et al., 2007). The 2VO model has shown that neuronal function is directly affected by mitochondrial bioenergetic abnormalities, which may trigger the onset of VD (Du et al., 2013). CCI is difficult to diagnose because it rarely occurs by itself and frequently cooccurs with other brain lesions (Zhao and Gong, 2015). A summary of drug treatment mechanisms is presented in Table 1.

**TABLE 1:** Specific performance of CCI therapeutic drugs.

Treatment	The target/pathway	Mechanism	Results	References
URB597	BNIP3, Beclin-1/Bcl-2 complex	Inhibition of impaired autophagic degradation and disruption of the Beclin-1/Bcl-2 complex, thereby severing mitophagy required for BNIP3-Cyt c- and parkin.	It prevents abnormal hyperautophagy and mitophagy.	Su et al., 2018
NAD	Sirt1/PGC-1α pathway	Mitochondrial protection is associated with ROS inhibition via activation of the Sirt1/PGC-1α pathway.	It improved cognitive function and reduced neuroinflammation in CCI model *in vivo* and *in vitro*.	Zhao et al., 2021
Baclofen	Akt/ERK-Bcl2-Beclin-1 signaling pathway	The induced phosphorylation of ERK1/2 moderately increased the expression of Beclin-1. Activation of GABA_A_ receptors improves GABA_B_ receptor α1 subunit surface expression, leading to downregulation of CX43 and CX36 surface and mitochondrial expression.	Enhancing cytoprotective autophagy can improve neuronal damage and cognitive impairment induced by CCI.	Liu et al., 2015
CIHH pretreatment	mitoK_ATP_	It can up-regulate the expression of Kir6.2 and SUR1 protein in mitochondria of hippocampal CA1 region and inhibit Cyt c-induced apoptosis.	It can improve the learning and memory dysfunction and DND in hippocampal CA1 region induced by ischemia.	Zhang et al., 2016
FMT and SCFAs	Histone demethylation acetylase (HDACs)	Normalization of mitochondrial membrane potential, reduction of ROS accumulation, and enhancement of mitochondrial ETC and oxidative phosphorylation.	Restore hippocampal mitochondrial function to improve cognitive dysfunction and treat colonic dysfunction.	Su et al., 2022
Carfilzomib	BNIP3L	BNIP3L degradation is prevented by inhibition of the ubiquitin-proteasome pathway.	Rescue the defect of mitophagy to prevent and reduce ischemic brain injury.	Wu et al., 2021
Butylphthalide	/	The activity of SOD in hippocampal mitochondria of rats increased, the content of malondialdehyde decreased, and the activity of ATPase increased.	The ability of learning and memory was significantly improved, and the degree of mitochondrial ultrastructure damage was further confirmed by pathology.	Gao, 2009
Pinocembrin	/	Its protective effects on components of the mitochondrial respiratory chain/oxidative phosphorylation system involve complex I activity, cytochrome oxidase expression, and the source of reactive oxygen species.	Long-term administration can improve cognitive dysfunction induced by cerebral hypoperfusion in rats.	Guang and Du, 2006
Rapamycin	PI3K/AKT/mTOR	The expression of mitophagy-related proteins was up-regulated, which could inhibit the overexpression of PI3K, AKT and mTOR.	Activation of mitophagia, in turn, prevents mitochondrial dysfunction and neuronal apoptosis, and ultimately improves brain injury and cognitive impairment.	Zheng et al., 2021
Endocannabinoid system	JNK	Enhanced the selective JNK inhibitor SP60012 and blocked JNK-dependent Bcl-2 signaling-induced neuronal apoptosis.	It improves mitochondrial membrane dysfunction and regulates neuronal survival.	Su et al., 2015
FTY720	Sirt3-independent pathway	The levels of pro-inflammatory cytokines and Iba-1 positive cells were decreased; after treatment, malondialdehyde level was decreased, ATP content was increased, and ATP synthase activity in hippocampus was up-regulated.	Improved memory performance, reduced neuroinflammation, and alleviated mitochondrial dysfunction, but had no effect on the reduction in Sirtuin-3 activity after CCI induction.	Zhang et al., 2020
Zuogui pill	/	Improved mitochondrial respiratory chain enzyme complex IV (COX) enzyme activity levels.	It can improve mitochondrial respiratory function, protect cell function and reduce ROS accumulation, thereby alleviating oxidative stress injury after CCI.	Yu et al., 2014
Naoxin’an capsule	CREB/PGC-1α signaling pathway	It significantly increased the activities of complex I, III and IV of mitochondrial respiratory chain and the activities of pyruvate dehydrogenase and α-ketoglutarate dehydrogenase in rats.	It can improve mitochondrial structure and function, increase mitochondrial membrane potential in brain tissue, and reduce oxidative damage caused by excessive ROS release.	Feng et al., 2022
Xiaoxuming decoction	/	Oxidative phosphorylation was increased, mitochondrial membrane potential was increased, and mitochondrial membrane swelling was reduced.	It alleviates mitochondrial dysfunction and structural damage caused by ischemia and hypoxia.	Wang Y. H. et al., 2012
Baicalein	/	There were improvements in membrane potential levels, oxidative phosphorylation processes, degree of mitochondrial swelling, Bcl-2/Bax ratio, and cytochrome c release.	It alleviates cognitive and motor impairment and reduces the production of mitochondrial reactive oxygen species.	He et al., 2009
Shenma Yizhi decoction	AMPK/PPARα/PGC-1α/UCP2 signaling pathway	The activities of SOD, GSH-Px and glutathione in serum were increased, and the content of malondialdehyde was decreased. In addition, the mRNA and protein expression levels of AMPK, PPARα, PGC-1α, UCP2 and ATP5A were reversed.	To improve mitochondrial structure and energy metabolism, thereby alleviating vascular cognitive impairment.	Sun et al., 2021
Bushen-Yizhi formula	/	It also reduces the occurrence of apoptosis and abnormal amyloid deposition and accumulation, and inhibits oxidative stress damage activated by abnormal and excessive mitochondrial autophagy in the hippocampus.	It can improve the cognition and memory ability of 2VO rats.	Xiao et al., 2023

### 4.1. Fecal microbiota transplantation (FMT) and short-chain fatty acids (SCFAs)

Short-chain fatty acids (SCFAs) produced by bacteria include acetate, propionate, and butyrate. These SCFAs can penetrate the blood-brain barrier and have a considerable impact on the brain due to their effects on numerous neuronal functions and gut-brain signaling pathways. FMT and SCFAs significantly altered Ndufb2 and Atp5mc1 levels, indicating that electron transport chain (ETC) complexes I and V are the main sites for the regulation of oxidative phosphorylation. FMT and SCFAs alleviate mitochondrial dysfunction by increasing acetate, acetyl-CoA, and ATP contents, as well as the activities of complexes I and V of mitochondrial ETC (Su et al., 2022).

### 4.2. Carfilzomib

Carfilzomib is a proteasome inhibitor that is used to treat multiple myeloma. It forms a covalent irreversible bond with the LMP2 and LMP7 catalytic subunits of the 20S proteasome, which are two intracellular receptors (Sin et al., 1999). Carfilzomib prevents defects in BCL/adenovirus E1B interacting protein 3-like (BNIP3L) degradation and mitophagy deficiency (Wu et al., 2021). Defective mitophagy caused by BNIP3L deletion has significant implications for ischemic neuronal injury. This is because restored BNIP3L has been observed to reduce cerebellar infarct volume, alleviating ischemic brain injury (Wu et al., 2021).

### 4.3. Butylphthalide

Butylphthalide is a chemical component of celery oil. Superoxide dismutase (SOD) activity increased, malondialdehyde levels decreased, and ATPase activity increased in the hippocampal mitochondria of CCI rats after therapy, significantly improving learning and memory. Pathological results provided additional evidence that injection reduced mitochondrial ultrastructural destruction. Butylphthalide injection has a protective effect on the structure and function of mitochondria in brain tissue, which may be related to its influence on mitochondrial oxidative damage and energy metabolism dysfunction (Gao, 2009).

### 4.4. Pinocembrin

Pinocembrin is a flavonoid found in propolis that can potentially strengthen the central nervous system. A decrease in transmembrane potential during hypoxia greatly affects mitochondrial function, producing excessive ROS (Nohl et al., 2005). In animal experiments, the expression of Cyt c oxidase in the hippocampus of rats in the 2VO group decreased significantly; meanwhile, mitochondrial membrane potential levels decreased. Pinocembrin significantly reversed these phenomena (Guang and Du, 2006). Cyt c oxidase is a metabolic indicator of neuronal oxidative activity; therefore, this raises the possibility that pinocembrin shields the rat’s brain mitochondria. In addition, pinocembrin can greatly reduce the degree of mitochondrial swelling, increase the mitochondrial membrane potential, and protect the mitochondrial structure and ROS production, which may explain why pinocembrin protects mitochondria from oxidative stress (Guang and Du, 2006).

### 4.5. Rapamycin

Rapamycin is a popular allosteric mTOR inhibitor that binds directly to the mTOR complex and promotes autophagy in several eukaryotes. PINK1, Parkin, and LC3B expression levels have been reported to increase after rapamycin treatment in animal studies, stimulating mitophagy and preventing mitochondrial dysfunction and neuronal apoptosis. Together with experimental treatment control of MHY1485 (an mTOR activator) and the initial notion that the mTOR pathway increases autophagy, it also affects the expression of PI3K, AKT, and mTOR (Bartolomé et al., 2017). These findings suggest that rapamycin exerts its neuroprotective effects by suppressing the PI3K/AKT/mTOR signaling pathway, which increases autophagy (Zheng et al., 2021).

### 4.6. Endocannabinoid system

The cannabinoid receptor agonist WIN55212-2 (WIN) and the fatty acid amide hydrolase inhibitor URB597 were administered to counteract the effects of CCI on JNK phosphorylation, lowering the Bcl-2/Bax ratio and caspase-3 activation, all of which are involved in controlling neuronal survival. Moreover, WIN and URB597 inhibit neuronal death induced by JNK-dependent Bcl-2 signaling and improve mitochondrial membrane dysfunction by increasing the selective JNK inhibitor SP600125 (Su et al., 2015).

### 4.7. FTY720

In 2010, the US Food and Drug Administration approved FTY720, a sphingosine-1-phosphate receptor agonist with potent anti-inflammatory properties, as the first oral medication for the treatment of multiple sclerosis (Wang et al., 2020). Moreover, recent studies have shown that it effectively reduces mitochondrial dysfunction and spatial memory impairment (Wang et al., 2020). FTY720 protects the brain from damage by lowering oxidative stress and neuroinflammation and enhancing synaptic function. According to a study in 2VO animals, FTY720 can improve hippocampal mitochondrial function and enhance ATP synthase activity. ATP levels and ATP synthase activity in the hippocampus are increased, suggesting that FTY720 could reduce CCI-induced mitochondrial dysfunction (Zhang et al., 2020). However, p62 expression, which is crucial for the transfer of ubiquitylated substrates to autophagosomes, and SIRT3, the primary regulator of mitochondrial activity, did not show an effect after the intervention (Zhang et al., 2020).

### 4.8. Traditional Chinese medicine

Traditional Chinese medicine has been reported to improve the activity of the ETC complex, decrease calcium overload following excitability toxicity, and restore the self-regulation function of mitochondria by focusing on mitochondrial dysfunction. This preserves the integrity of mitochondrial structure and function, promotes the reconstruction of energy metabolism, and ultimately improves brain injury and cognitive impairment (Wang et al., 2023). For example, the Zuogui pill and Naoxin capsule improve mitochondrial structure and function and reduce ROS accumulation by improving mitochondrial respiratory chain enzyme complexes (Yu et al., 2014; Feng et al., 2022). Xiaoxuming decoction and baicalein have significantly improved oxidative phosphorylation and mitochondrial membrane potential (He et al., 2009; Wang Y. H. et al., 2012). The Shenma Yizhi decoction and Bushen-Yizhi formula can improve mitochondrial dysfunction by regulating the expression levels of various proteins (Sun et al., 2021; Xiao et al., 2023).

## 5. Prospects

The pathogenesis of persistent cerebral ischemia is complex. One of the main reasons for brain injury and aberrant alterations in brain function caused by prolonged cerebral ischemia is the impairment of brain energy metabolism. Increased free radical production, oxidative stress damage, and altered mitochondrial structure and function contribute significantly to the pathophysiology of CCI (Zhou et al., 2021). Therefore, the significance of mitochondrial dysfunction in CCI has received considerable attention, and it is crucial to investigate changes in mitochondrial structure and function to better understand the effect of medications on chronic cerebral ischemia.

Few clinical studies on pharmacological therapy for CCI are currently available, with the majority focusing on the development of new medications to treat cerebral ischemia-reperfusion injury. Most medications play a limited clinical role in the management of persistent cerebral ischemia (Lana et al., 2014; Kim et al., 2016; Yan et al., 2022). According to recent studies, URB597 blocks the Parkin route to restrict mitophagy, NAD stimulates the PPAR pathway to prevent ROS release, and Baclofen-induced ERK1/2 phosphorylation can accelerate cytoprotective autophagy. Whether there are any further pathways for the treatment of CCI remains unknown. Therefore, it is important to understand the mechanisms, identify newer and more potent therapeutic targets, introduce pharmaceuticals into trials in humans for clinical evaluation, and improve the efficacy and safety of medications.

## Author contributions

ZC and MZ: conception and design. ZC and XY: administrative support. MY: provision of study materials, collection, and assembly of data. MY, PF, and MW: data analysis and interpretation. ZC: revised the final version. All authors contributed in manuscript writing and approved the final version of manuscript.
